# Conservative Management for Urethral Foreign Body: A Case Report of an Adolescent Boy With Repeated Events

**DOI:** 10.3389/fped.2021.691778

**Published:** 2021-07-26

**Authors:** Adam Bezinque, Ahmad Mohamed, Jeffrey White, Katie Canalichio, Dennis Peppas, Eran Rosenberg

**Affiliations:** ^1^Department of Urology, University of Louisville, Louisville, KY, United States; ^2^Department of Pediatric Urology, Norton Healthcare, Louisville, KY, United States

**Keywords:** urethral foreign body, void, observation, psychiatric illness, urethra

## Abstract

Placing foreign bodies into the urethra is not a common occurrence in the general population. Patients self-insert foreign bodies for a multitude of reasons such as sexual gratification, secondary gain, and psychiatric illness. From our own experience and what has been reported in the literature, there is a wide variability in the type of objects that patients place into the urethra. We report a unique case of a 17-year-old adolescent boy with repeated foreign body insertions into the urethra over a 1-year period. This patient suffers from significant psychiatric illness. Due to the number of events in this past year, we initiated a conservative observational approach that contrasts the traditional invasive protocol to treat with endoscopic removal. This management has proven to be successful in his case and can be replicated in other scenarios after careful consideration of the clinical presentation.

## Introduction

Urethral foreign bodies are infrequently encountered in the urology practice. Each case is unique given that these individuals have very personalized reasons to insert an object in their urethra. Psychiatric illness is known to be a main cause as well as autoerotic stimulation (1–4). We report a patient that has a significant psychiatric history including bipolar depression, oppositional defiant disorder, and reactive attachment disorder. He has had 20 visits in the past year to our emergency department for urethral foreign body insertion prompting a conservative course of management.

## Case Description

**Figure 1:**
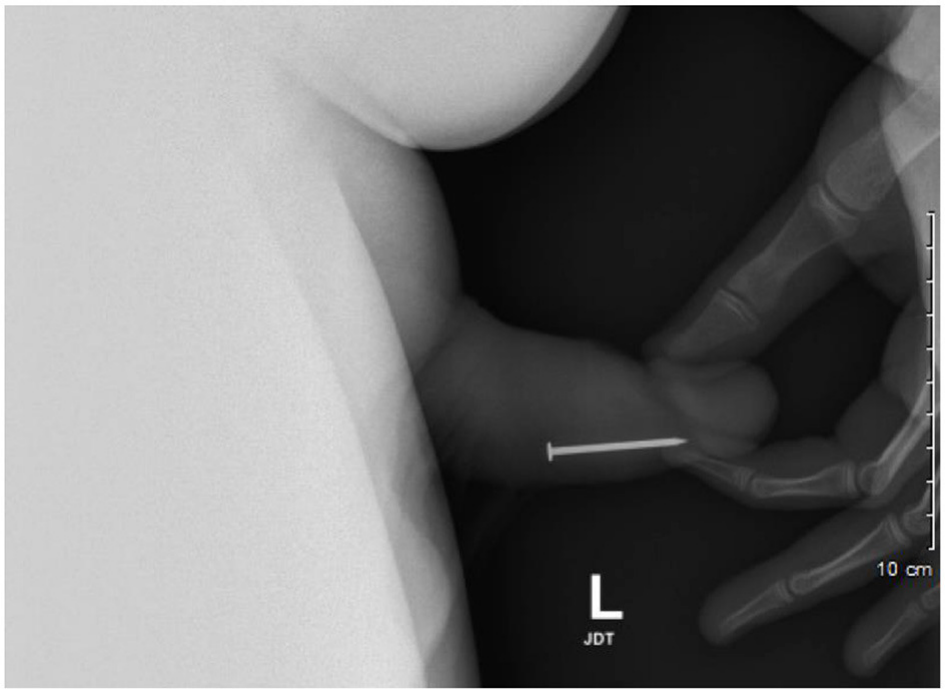
Lateral view pelvic X-ray identifies nail in distal penile urethra, which requires endoscopic removal.

A 17-year-old adolescent boy with significant psychiatric history and prior history of self-insertion of foreign bodies per urethra first presented to our institution after inserting a nail per urethra in an act of self-harm. This was identified on plain film X-ray (Figure 1). The patient was brought to the operating room and placed under general anesthesia, and the foreign body was removed using a 19fr cystoscope and flexible grasper.

Since his initial encounter, he had a total of 20 emergency department visits for urethral foreign body, and nine of these encounters required endoscopic removal. He did place several foreign bodies per urethra during one admission. In total, he was able to expel a urethral foreign body 13 times, thereby preventing surgical intervention or external manipulation at bedside. He also never required admission for trial of passage.

**Figure 2:**
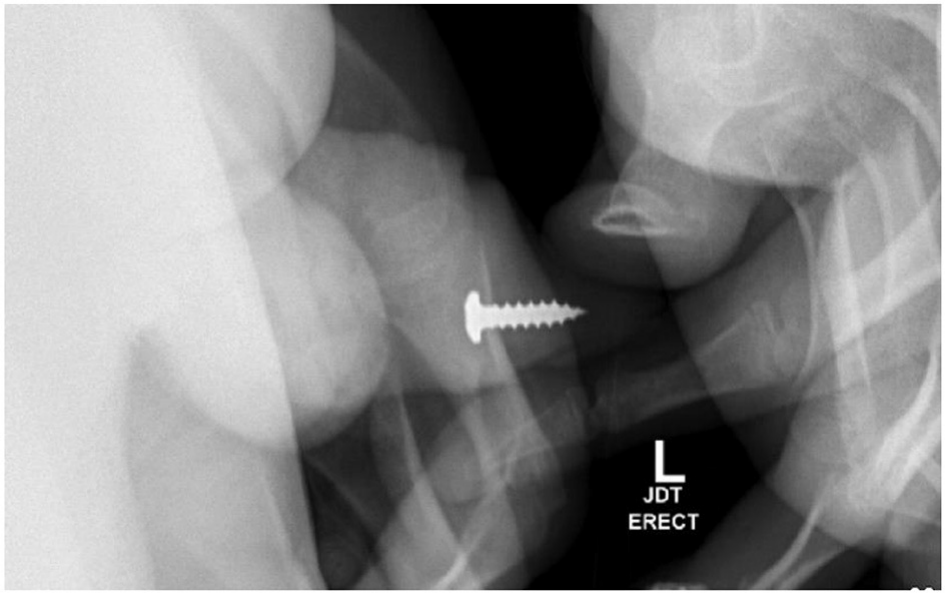
Pelvic X-ray identifies metal screw in distal penile urethra, which was removed at bedside using a hemostat.

Several months later, he presented with self-insertion of a metal screw into his urethra in addition to hydroxyzine ingestion. During this presentation, a foreign body was palpable in the distal penile urethra, and a plain film X-ray (Figure 2) identified the location of the screw. His symptoms primarily consisted of urinary retention and penile pain.

**Figure 3:**
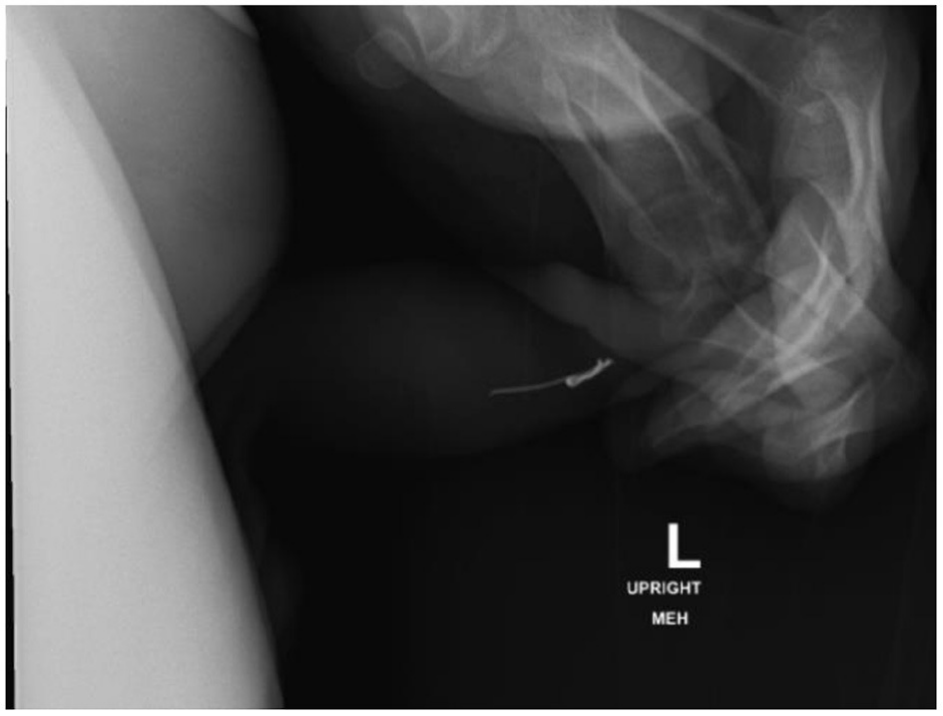
Lateral view pelvic X-ray identifies metal wire from hospital face mask in distal penile urethra, which was voided spontaneously.

**Figure 4:**
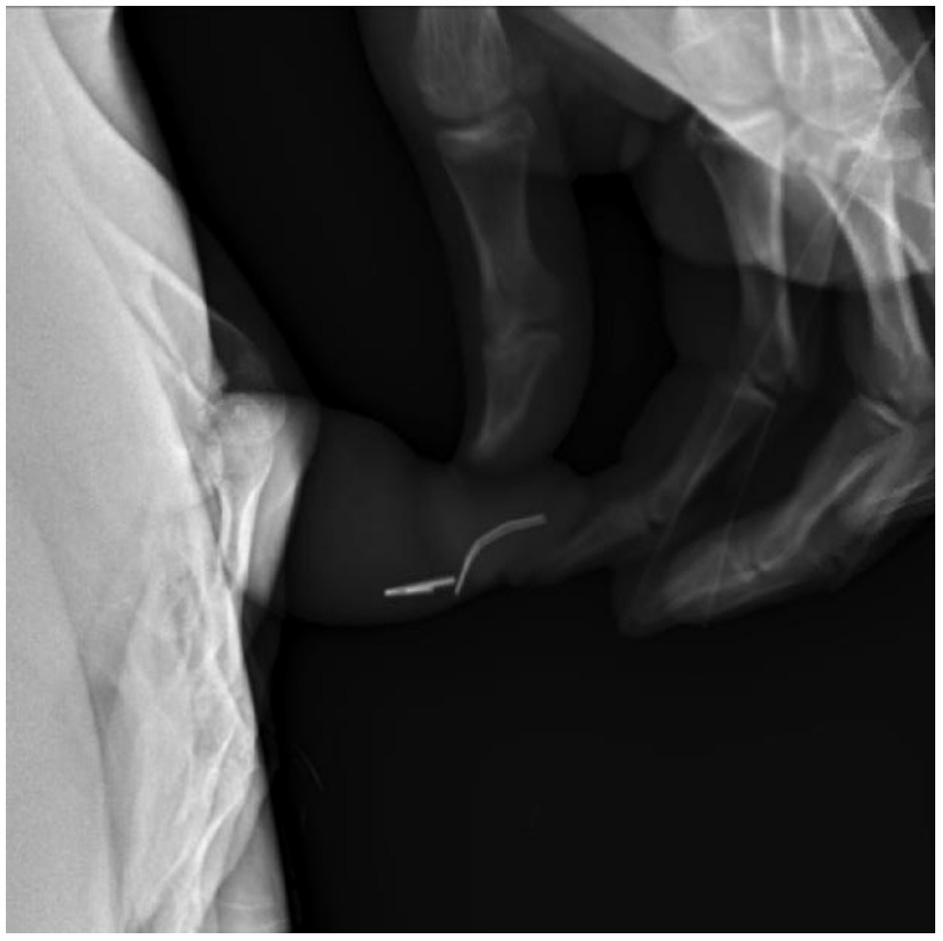
Lateral view pelvic X-ray identifies staples in distal penile urethra, which requires endoscopic removal.

In review of his history of foreign bodies per urethra, he has been able to void a piece of plastic coffee lid, small construction nails, metal wire from hospital face mask (Figure 3), and a plastic fork tong. Objects that required endoscopic removal included staples (Figure 4), assorted pieces of plastic, and larger nails. If the object appeared sharp and/or proximally placed in the urethra, we favored endoscopic removal to prevent damage to the urethra. After several trips to the OR for endoscopic removal, we started to identify urethral scarring from repeated trauma.

We are aware that he has a higher than normal bladder capacity for his age group. After several of his prior trips to the emergency department, he would typically present retaining up to 1 L of urine without showing signs of discomfort. The screw was located in the distal urethra and positioned so the blunt end would be expelled first. We continued conservative observational management with trial of void for the patient to expel the foreign body. Our indications to take him to the operating room included urinary retention with 1.0–1.5 L on bladder scan and/or severe uncontrolled pain. Eventually, he voided 1 L of urine with the foreign body without complication. He had no complaints of abdominal pain during this period of observation. Once he was able to demonstrate a second spontaneous void, there was no further urological intervention indicated other than a 5-day course of oral antibiotics. We have attempted to arrange follow-up for each visit. Unfortunately, his situation is particularly difficult given the fact that he is frequently admitted to the inpatient psychiatric facility and overall non-compliant with our recommendations. On each occasion, we had warned him of the expected long-term trauma to his urethra. It would be ideal to follow him in an office-based setting to evaluate his voiding habits, monitor his behavior, and perform a videourodynamic study.

## Discussion

Encountering a patient with a urethral foreign body is a rare and unique situation in the urology practice, but it is not a new problem. There are many reported cases in the literature pertaining to urethral foreign body insertion detailing a wide variety of objects and situations. Rahman et al. performed a retrospective analysis of patients with palpable foreign bodies in the urethra, which included 17 patients. The most common reasons for insertion among this cohort was psychiatric disorder, followed by intoxication and sexual gratification (1). Masturbation and sexual curiosity are the leading cause of penile injury by foreign bodies in adolescents (2, 3). Symptoms due to urethral foreign bodies commonly consist of dysuria due to mechanical urethritis, purulent urethral discharge, and hematuria. Larger objects raise the possibility of urinary retention and subsequent damage to the upper urinary tract (2, 4, 5).

The patient presented in our case has multiple psychiatric diagnoses and a significant number of repeated events of foreign body insertion. He has now accumulated 20 visits to the emergency department related to urethral foreign body insertion. Every measure has been taken to treat his psychiatric issues, but it is near impossible to prevent someone from choosing to insert a foreign body. He was initially taken to the operating room nine times in the past year for endoscopic removal of a foreign body, so we needed to employ more conservative measures when possible, which included attempts of milking out distal foreign bodies or using a hemostat at bedside. Eventually, we began the trial of passage with resulting success. Typically, he presented with urinary retention, and coupled with the number of events, he had developed a very large bladder capacity. We became comfortable with close observation, serial bladder scans, and trial of void for him to self-expel the foreign body. In this case, this has proven to be very successful. He voided foreign bodies on 13 separate occasions. Twice, we removed a foreign body at bedside utilizing lidocaine gel and a hemostat. Objects that he was able to void included pieces of plastic coffee lid, zip tie, dry wall nail, metal screw, metal piece from face mask, plastic fork prong, and staples. Location in the urethra also favored with our suggestion that objects in the distal penile urethra were more likely to pass with voiding. We have had luck with this patient voiding objects in the bulbar urethra as well. In the past seven times that he presented with urethral foreign bodies, he was able to void them on his own, thereby avoiding intervention in the operating room.

Traditional management of a urethral foreign body is endoscopic retrieval utilizing grasping instruments such as forceps, snares, and stone retrieval baskets. If endoscopy is unsuccessful, patients may require external urethrotomy to retrieve the object. If left untreated, urethral foreign bodies can lead to infection, stones, diverticula, and fistula formation (1, 2, 6). Most contemporary literature favor endoscopic management, and to our knowledge, there are few, if any, case reports detailing successful conservative management. Spontaneous expulsion has already been reported as far back as the 1800s by Poulet, but it was stated that this method is generally incapable of giving good results. Poulet also cited Lavallee performing external manipulation but described this as poorly regulated, dangerous, and only to be used in very exceptional cases (7). This can also be termed “milking” the urethra and has been utilized in many instances including retrieving a leech from the urethra (8). Palmer et al. performed a 15-year retrospective chart review of 27 patients with self-inserted urethral foreign bodies, and in 7 (20%) cases, the patients were able to pass the foreign bodies during voiding (9). They also provided a good algorithm for the evaluation and management of urethral foreign bodies. We would like to offer a trial of passage or manual extraction for urethral foreign bodies that have the characteristics that would favor passage such as small size (<1 cm), palpable, mobile, blunt, in distal penile urethra, favorable orientation confirmed on X-ray, and without gross hematuria or other significant symptoms. Additional methods can be employed to facilitate spontaneous passage with a bolus of intravenous fluids, topical lidocaine gel, and pain medication.

Our case report is strengthened by the fact that our patient had 20 documented instances of inserting a foreign body into his urethra. In addition, of these 20 instances, there was success with conservative observational management on more than one occasion. Unfortunately, there is only one patient involved with our case, and we have yet to study this approach with other patients.

We present our case to exemplify that spontaneous expulsion of urethral foreign bodies remains a valuable option prior to performing invasive maneuvers. It is important to bear in mind the object's shape, size, and location, in addition to the patient's overall clinical presentation including history of prior events and the need for close observation in such cases.

## Data Availability Statement

The original contributions presented in the study are included in the article/supplementary material, further inquiries can be directed to the corresponding author/s.

## Ethics Statement

Written informed consent was obtained from the relevant individual(s), and/or minor(s)' legal guardian/next of kin, for the publication of any potentially identifiable images or data included in this article.

## Author Contributions

All authors contributed to manuscript revision, read, and approved the submitted version.

## Conflict of Interest

The authors declare that the research was conducted in the absence of any commercial or financial relationships that could be construed as a potential conflict of interest.

## Publisher's Note

All claims expressed in this article are solely those of the authors and do not necessarily represent those of their affiliated organizations, or those of the publisher, the editors and the reviewers. Any product that may be evaluated in this article, or claim that may be made by its manufacturer, is not guaranteed or endorsed by the publisher.
